# Photocatalytic Activity of Zn_*x*_Mn_3−*x*_O_4_ Oxides and ZnO Prepared From Spent Alkaline Batteries

**DOI:** 10.3389/fchem.2020.00661

**Published:** 2020-08-13

**Authors:** Lorena Alcaraz, Eva Jiménez-Relinque, Lorenzo Plaza, Irene García-Díaz, Marta Castellote, Félix A. López

**Affiliations:** ^1^National Center for Metallurgical Research (CENIM-CSIC), Madrid, Spain; ^2^Institute of Construction Science, “Eduardo Torroja” (IETcc-CSIC), Madrid, Spain

**Keywords:** Zn/Mn binary oxide, ZnO, spent alkaline batteries, photocatalysis, UV and Vis irradiation

## Abstract

Oxides with Zn_*x*_Mn_3−x_O_4_ stoichiometries and ZnO were synthesized from the “black mass” material recovered from spent alkaline batteries. The oxides were characterized by XRF, XRD with Rietveld refinement, SEM, and TEM methods. Optical characterization included diffuse reflectance (DRS) and photoluminescence (PL) measurements. ZnO presented a clear band edge in the UV region, and PL signals were detected. The Zn/Mn oxides showed strong absorption in the UV region and a continuous absorption band in the Vis-IR regions. There is a non-detected PL signal due to excited charges being trapped on sub-band energy states and/or transfer by non-radiative paths. Photocatalytic activity under both irradiation conditions was evaluated using the resazurin dye test, terephthalic acid fluorescence probe method, and NO_x_ air purification evaluation. In the three photoactivity tests, ZnO performed well under both UV and Vis irradiation, whereas no evidence of any appreciable photocatalytic activity was observed for the Zn/Mn oxides. The results are discussed in terms of the findings of previously reported optical measurements.

## Introduction

An unfortunate accompaniment to the tremendous and continuous development of society is the problem of serious environmental pollution. For example, alkaline batteries serve a valuable purpose as portable energy sources for many electronic devices. However, of the more than 80 billion spent alkaline batteries generated annually, <50% are usefully recycled, while the rest are discarded in landfills as hazardous wastes (Bernardes et al., 2004). The materials in these batteries include manganese dioxide, zinc, and other heavy metals that can seep out and negatively affect the environment (Bernardes et al., 2004). This potential danger, among others, makes the recycling of batteries an important facet of environmental protection. To date, several routes for recycling spent alkaline batteries involving hydro- and pyrometallurgical processes have been patented. Previous studies have reported that binary Zn/Mn oxides with varying stoichiometries (Zn_*x*_Mn_3−x_O_4_) (Alcaraz Roma et al., 2018), as well as zinc oxide (ZnO) (Cebriano et al., 2017; López et al., 2017), can be obtained from the “black mass” waste that is generated after the disassembly and mechanical separation of spent alkaline batteries.

In addition to recycling to protect our environment, contaminant remediation is of particular importance nowadays. In this sense, new processes to decompose environmental pollutants based on photocatalytic reactions are being assessed. The photocatalytic process begins when a photon with enough energy (*hυ* ≥ band gap) reaches the surface of the photocatalyst, exciting an electron from the valence (VB) to the conduction band (CB) and leading to the formation of an electron–hole (e^−^/h^+^) pair. These generated species migrate to the surface of the photocatalyst where, in the presence of adsorbed water and oxygen molecules, they form reactive oxygen species, mainly OH^·^ and ${\text{O}}_{2}^{\cdot PICT\;-}$, which can participate in redox reactions to eliminate adsorbed pollutants. Among these harmful toxins, nitrogen oxides (NO and NO_2_, both considered NO_x_) are considered major atmospheric pollutants that negatively affect the health of animals, plants, and humans (Lasek et al., 2013). These compounds are very harmful and poisonous gases that are emitted primarily from combustion. Their photocatalytic oxidation (NO → HNO_2_ → NO_2_ → ${\text{NO}}_{3}^{-}$) has been previously reported (Jimenez-Relinque et al., 2015).

Among the available photocatalysts, nano-sized titanium dioxide (TiO_2_) is the most widely used because of its chemical stability, good optical transparency, high refractive index, low cost, and non-chemical toxicity. Nevertheless, its limited visible light absorption, together with its price, are considered obstacles to its wider application (Jimenez-Relinque et al., 2015; Nava-Núñez et al., 2020). Thus, the design and development of low-cost, high-performance catalysts that are preferably sustainable and active across the entire solar spectrum should be key objectives. Several researchers have evaluated the photocatalytic activities of MnO_2_-TiO_2_ (Li et al., 2008a) and MnO_2_-ZnO (Pung et al., 2016) mixtures. However, lower photocatalytic activity was observed for ZnO and TiO_2_ in the presence of MnO. Combining both aspects, oxides prepared from spent batteries have been tested as photocatalyst materials to degrade organic contaminants in water (Alcaraz et al., 2019), proving high photocatalytic activity under UV irradiation for mixed Zn/Mn oxides.

In this study, oxides with Zn_*x*_Mn_3−x_O_4_ stoichiometries and ZnO were synthesized from the black mass obtained from spent alkaline batteries by acid leaching followed by a selective precipitation procedure. The materials were characterized in terms of their chemical compositions, structures, and optical properties. The photocatalytic performance and efficiencies of the prepared samples under UV and visible irradiation were investigated in three different photoactivity tests.

## Materials and Methods

### Preparation of Zn_x_Mn_3-x_O_4_ Oxides

Three Zn_*x*_Mn_3−x_O_4_ oxides were prepared from the black mass produced from spent alkaline batteries by a two-step procedure that included acid leaching of the black mass followed by selective precipitation from the resulting solution in basic medium. The black mass used as the starting material was provided by a Spanish company dedicated to the mechanical recycling of spent batteries. Different amounts of black mass (100–300 g) were leached in MilliQ water (250 ml), H_2_O_2_ (250 ml, PanReac®), and HCl (500 ml, PanReac) at room temperature for 1 h. The mixtures were filtered through a Millipore Holder filter under pressure (7 bar). Subsequently, the collected solutions were selectively precipitated by adding NaOH (6 M) until pH 12–14 was achieved. The generated brown precipitates were filtered and dried at 353 K. The final solids obtained from solid/liquid ratios of 100, 200, and 300 g·L^−1^ are designated L100, L200, and L300, respectively. To prepare the ZnO sample, after the described basic precipitation step, HCl was slowly added until the pH decreased to 8–9. This generated ZnO as a whitish precipitate that was filtered and dried.

### Characterization

The elemental compositions of the samples were determined by X-ray fluorescence (XRF) analysis using a PANalytical Axios wavelength dispersive spectrometer (4 kW). Structural characterization was performed by X-ray diffraction (XRD) using a Siemens D5000 diffractometer with CuKα radiation. Powder diffraction data were analyzed by the Rietveld method using version 4.2 of the Rietveld analysis program TOPAS (Bruker ASX) and crystallographic information for the different phases obtained from a database for inorganic compounds, Pearson's Crystal Structure (Villars and Cenzual, 2009). The morphological characterization of the samples was carried out by scanning electron microscopy (SEM) using a field-emission SEM instrument (JEOL JSM 7600) and by transmission electron microscopy (TEM) using a JEOL JEM 2100 instrument. For the SEM observations, the powder samples were placed on an adhesive conductive carbon disk and gold coated. In the case of the TEM measurements, powder samples were dispersed in *n*-butanol and drops of the corresponding suspensions were deposited on carbon-coated copper grids. The UV–Vis diffuse reflectance spectra (DRS) of all samples were acquired on a Shimadzu UV-2600 spectrophotometer with an integrating sphere. Absorption spectra were obtained from the reflectance data using the Kubelka–Munk equation (Pradarelli et al., 2017) (Equation 1) in the range 200–850 nm:

(1) $$\begin{array}{r} F\left(x\right)=\;\frac{{\left(1-R\right)}^{2}}{2R} \end{array}$$

Photoluminescence (PL) spectra were obtained on a PerkinElmer LS-55 fluorescence spectrometer with excitation at 315 nm. The scanning region encompassed the range of 350–550 nm.

### Photocatalytic Activity Tests

#### Resazurin Dye Test

The photocatalytic activity of the as-prepared samples was evaluated using the resazurin (Rz) ink test. The effectiveness of resazurin (Rz) dye as an indicator ink for photocatalytic activity was previously demonstrated by the Mills group (Mills et al., 2005, 2006, 2013, 2014). Upon irradiation (*h*υ ≥ band gap energy) of the photocatalyst surface, photogenerated electrons rapidly reduce blue Rz to pink resorufin (Rf) in the first stage. Rf can be reduced to its colorless counterpart, dihydroresorufin (HRf). Simultaneously, the photogenerated holes oxidize glycerol to glyceraldehyde, which acts as a hole trap to prevent electron–hole recombination. Rz and Rf exhibit characteristic absorption peaks at 600 and 570 nm, respectively. The color/absorbance change provides proof of the photocatalytic activity of the tested photocatalyst.

**Figure 1:**
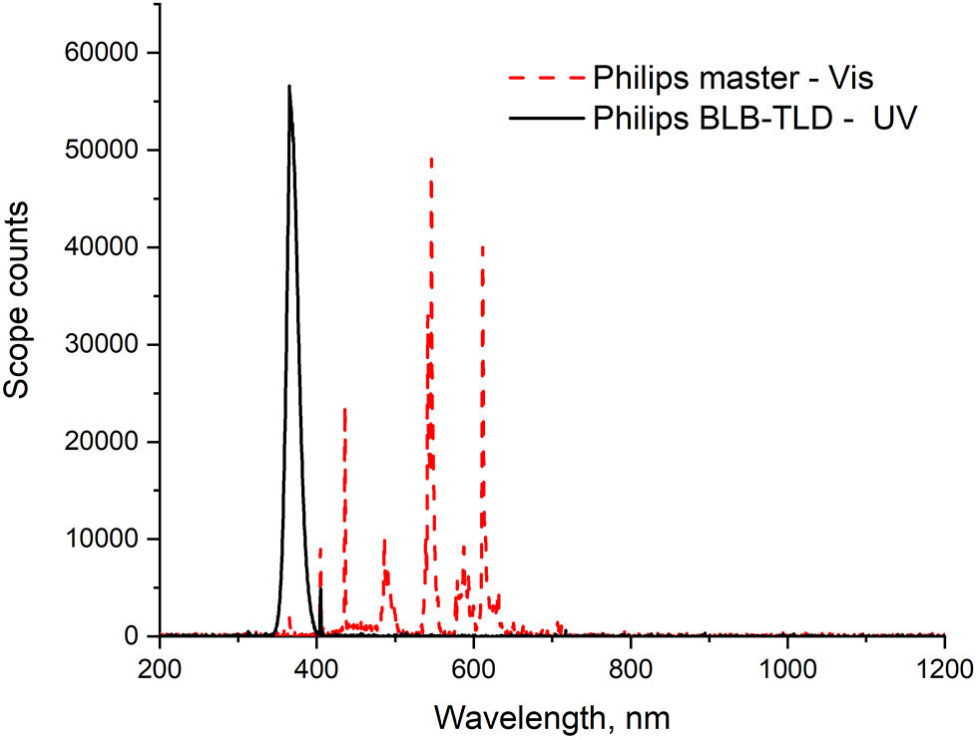
Spectral irradiance profiles of the fluorescent light sources used.

The Rz test solution was prepared by mixing the dye (1 ppm) and glycerol (0.04 g) in water (200 ml). In the photocatalytic activity assessment, each powder (0.05 g, except for ZnO (0.004 g), which exhibited higher photocatalytic activity) was magnetically stirred in the Rz solution (10 ml) in a glass vial. The suspensions were kept in the dark with magnetic stirring until the adsorption–desorption equilibrium between the sample and Rz was reached. Then, the suspensions were irradiated while continuously stirring. Thereafter, the solids were separated by centrifugation and the UV–Vis absorption spectra of the supernatant solutions were measured under different irradiation times using a Shimadzu UV-2600 spectrophotometer. Irradiation was conducted using two Philips Actinic BL TL-D 15W UV fluorescence lamps, which emitted photons in the wavelength range of 350–400 nm with an optimum wavelength of 365 nm. The light spectrum of the lamps is shown in Figure 1. To determine photocatalytic efficiency, the evaluation of dye degradation was only performed under UV light because the dyes are inappropriate for evaluating the photocatalytic activity. Operations under visible light are severely limited by a variety of factors, most of which are related to the presence of a second mechanism, sensitization (Wu et al., 1998; Rochkind et al., 2015; Jimenez-Relinque and Castellote, 2018).

#### Terephthalic Acid (TA) Fluorescence (FL) Probe: Hydroxyl Radical (OH_·_) Generation

The amount of OH_·_ formed by each sample was detected using a terephthalic acid (TA) probe. TA reacts readily with OH_·_ photogenerated by the photocatalyst to produce a highly fluorescent product, 2-hydroxyterephthalic (TAOH, λ_exc_ = 315 nm; λ_em_ = 425 nm) (Equation 2). The amount of TAOH measured is consequently proportional to the OH_·_ produced by the material tested.

(2) $$\begin{array}{r} TA+OH_{\cdot }\;\to \;TAOH \end{array}$$

The TA probe solution was prepared using a previously defined optimum formulation (TA, 2 mM neutralized NaOH, 0.02 M, pH 12.4 ± 0.2) (Jimenez-Relinque and Castellote, 2015). The test was performed using a suspension of each powder [0.05 g, except ZnO (0.004 g)], which exhibited higher photocatalytic activity] in the TA probe solution (10 ml) in a glass vial. The suspension was continuously stirred and irradiated for 0, 15, 30, 45, or 60 min using two UV fluorescence lamps, the Philips Actinic BL TL-D 15W and Philips Master TL-D Super 80 for UV or visible irradiation tests, respectively. The mean light intensity at the surface of the reaction solution was ~10 W/m^2^. Figure 1 shows the irradiance spectra of the two lamp types used.

The suspension was centrifuged to remove the catalyst powder, and the fluorescence intensity of the supernatant solution was measured using a Perkin-Elmer LS-55 fluorescence spectrophotometer with an excitation wavelength of 315 nm. The amount of TAOH generated was quantified from the calibration curve obtained by plotting the FL intensity at 425 nm against previously reported standard TAOH concentrations [FL (a.u.) = 143.66∙[TAOH] (μmol)] (Jimenez-Relinque and Castellote, 2015). The OH_·_ formation rate was calculated from the slope of the FL intensity–irradiation curve, assuming a trapping factor of 35% (Fang et al., 1996; Sahni and Locke, 2006). Further details on the experimental setup can be found in works by Sahni and Locke (2006) and Laplaza et al. (2017).

#### Nitrogen Oxide Removal Test (ISO 22197-1:2007)

The photocatalytic activities of as-prepared samples L300 and ZnO were also evaluated as specified in ISO standard 22197-1:2007: Nitrogen oxide removal test. These samples were selected because they showed better photoactivity performance in the Rz ink test (see section Resazurin Dye Test). The test gas (3 ± 0.15 L/min) was adjusted to an initial concentration of 1,000 ± 50 ppb NO in air at 25 ± 2.5°C and 50 ± 5% relative humidity (RH). Squares (5 × 5 cm^2^) of pressed powder were prepared using 100 mg of each sample and placed in the test chamber. After allowing the gas to flow into the reactor without irradiation for 30 min, the sample was irradiated for 30 min. Irradiation was subsequently interrupted and confirmed that the flow was switched to the original concentration of gas. The NO_x_ (NO + NO_2_) concentration in the gas was monitored with an Environment S.A. AC32M chemiluminescence analyzer. Efficiencies (η) for NO removal, NO_2_ formation, and total NO_x_ removal were determined separately. This analytical procedure is described in greater detail elsewhere (Jimenez-Relinque and Castellote, 2015; Jimenez-Relinque et al., 2015; Nava-Núñez et al., 2020). This test was performed under both UV and visible light irradiation. Two Philips BLB TL-D 15 W fluorescence tubes were used for UV tests. The visible light source consisted of two Philips Master TL-D Super 80 fluorescence tubes, emitting photons in the wavelength range of 406–700 nm. The average light intensity at the surface of the reaction solutions or pressed powders was ~10 W/m^2^ and 16,000 lux for the UV and Vis light tests, respectively. The light spectra for both lamp types are shown in Figure 1.

## Results and Discussion

### X-Ray Fluorescence (XRF)

The chemical compositions of the obtained powders are shown in Table 1. Samples L100, L200, and L300 are mainly composed of Mn and Zn, with other compounds present in minor amounts. It should be noted that the Mn content is higher in the L100 sample than in the L200 and L300 samples. In all cases, the amount of Mn is much higher than that of Zn. An opposite trend is observed for the Zn content. Following Mn and Zn, the wt% Na content is the next highest found in all analyzed samples. These results are related to the preparation method, in which NaOH was used to precipitate the Zn_*x*_Mn_3−x_O_4_ solids from the liquids. The chemical composition of the ZnO sample is >98 wt% Zn, despite the presence of minor elements.

**Table 1:** Chemical compositions of the obtained samples.

**Compound**	**L100**	**L200**	**L300**	**ZnO**
	**(wt%)**			
Na_2_O	1.64	3.40	6.83	–
MgO	0.20	–	0.21	0.22
Al_2_O_3_	0.11	0.24	0.1	0.55
SiO_2_	0.13	0.24	0.25	0.46
P_2_O_5_	0.93	0.99	1.00	–
Cl	0.71	1.40	2.48	0.25
CaO	0.43	0.41	0.47	0.25
TiO_2_	0.08	0.16	–	–
Cr_2_O_3_	0.04	0.12	–	–
MnO	86.20	73.57	62.26	0.02
Fe_2_O_3_	1.11	2.42	0.16	0.01
Co_3_O_4_	0.10	0.06	0.07	–
NiO	0.84	0.60	0.99	0.01
CuO	0.05	0.23	–	0.11
ZnO	7.17	15.03	24.92	98.09
SrO	0.05	0.06	0.04	–
Nb_2_O_5_	0.11	0.07	–	–
CdO	0.04	0.16	0.05	–

### Structural Characterization

**Figure 2:**
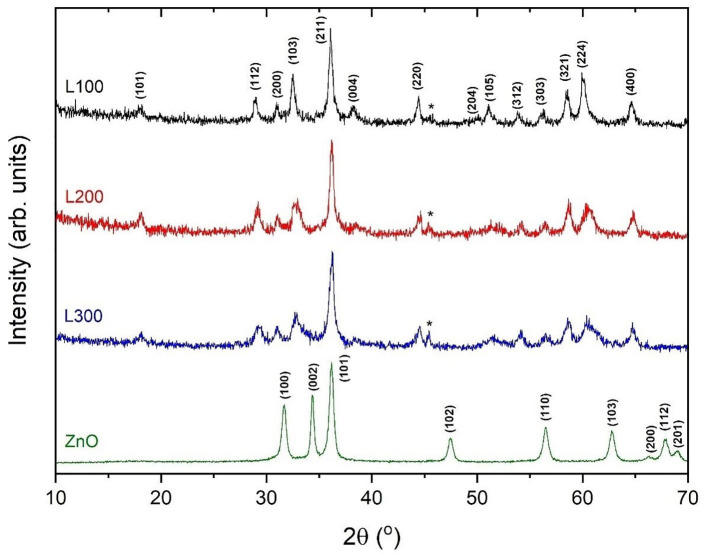
XRD patterns for the prepared samples.

The X-ray diffraction patterns of the prepared samples are shown in Figure 2. Among the L100–300 samples, the most intense reflection maxima can be indexed to a tetragonal symmetry with space group *I*4_1_*amd*, compatible with the spinel-type structure for ZnMn_2_O_4_ (JCPDS data card no. 24-1133). Some impurities related to the preparation method and attributed to the NaCl phase (marked in the figure with asterisks) are observed, in agreement with the XRF results. However, the impurity content is minor (<5% in all cases). To improve structural characterization, the X-ray diffraction data were refined by the Rietveld method (Rietveld, 1967); Table 2 summarizes the results. The analysis suggests different stoichiometries for the obtained samples according to the Zn_*x*_Mn_3−x_O_4_ formula. In agreement with the XRF results, the L100 sample exhibits a higher Mn content than the L200 and L300 samples. The calculated average crystallite size using the Scherrer equation (Equation 3) was 38, 43, and 55 nm for the L100, L200, and L300 samples, and 72 nm for ZnO sample. It can be observed that the average particle size increase with the Mn content is in good agreement with the results previously reported by other authors (Hadžić et al., 2016). In addition, the lattice parameters calculated from the refined XRD patterns are shown in Table 2. The cell parameters increase slightly with the Mn and Zn content in the samples, as previously reported for samples with similar stoichiometries (Nádherný et al., 2016). However, the *c*/*a* ratio remains constant in all cases, indicating that structural distortion does not occur.

(3) $$\begin{array}{r} D\left(nm\right)=\;\frac{0.89\lambda }{\beta cos\theta } \end{array}$$

In the case of the ZnO sample, all diffraction maxima can be indexed to the wurtzite-type structure of ZnO (JCPDS data card no. 36-1451) with hexagonal symmetry in the *P*6_3_*mc* space group. No other peaks attributable to secondary phases are observed, within the sensitivity of the experimental system.

**Table 2:** Suggested stoichiometries and lattice parameters for Zn_*x*_Mn_3−x_O_4_ samples obtained by Rietveld analysis.

**Sample**	**Stoichiometry**	***a* = *b* (Å)**	***c* (Å)**	***c*/*a***
L100	Zn_0.25_Mn_2.75_O_4_	5.766	9.393	1.61
L200	Zn_0.85_Mn_2.15_O_4_	5.756	9.266	1.61
L300	ZnMn_2_O_4_	5.758	9.258	1.61

### Morphological Characterization

**Figure 3:**
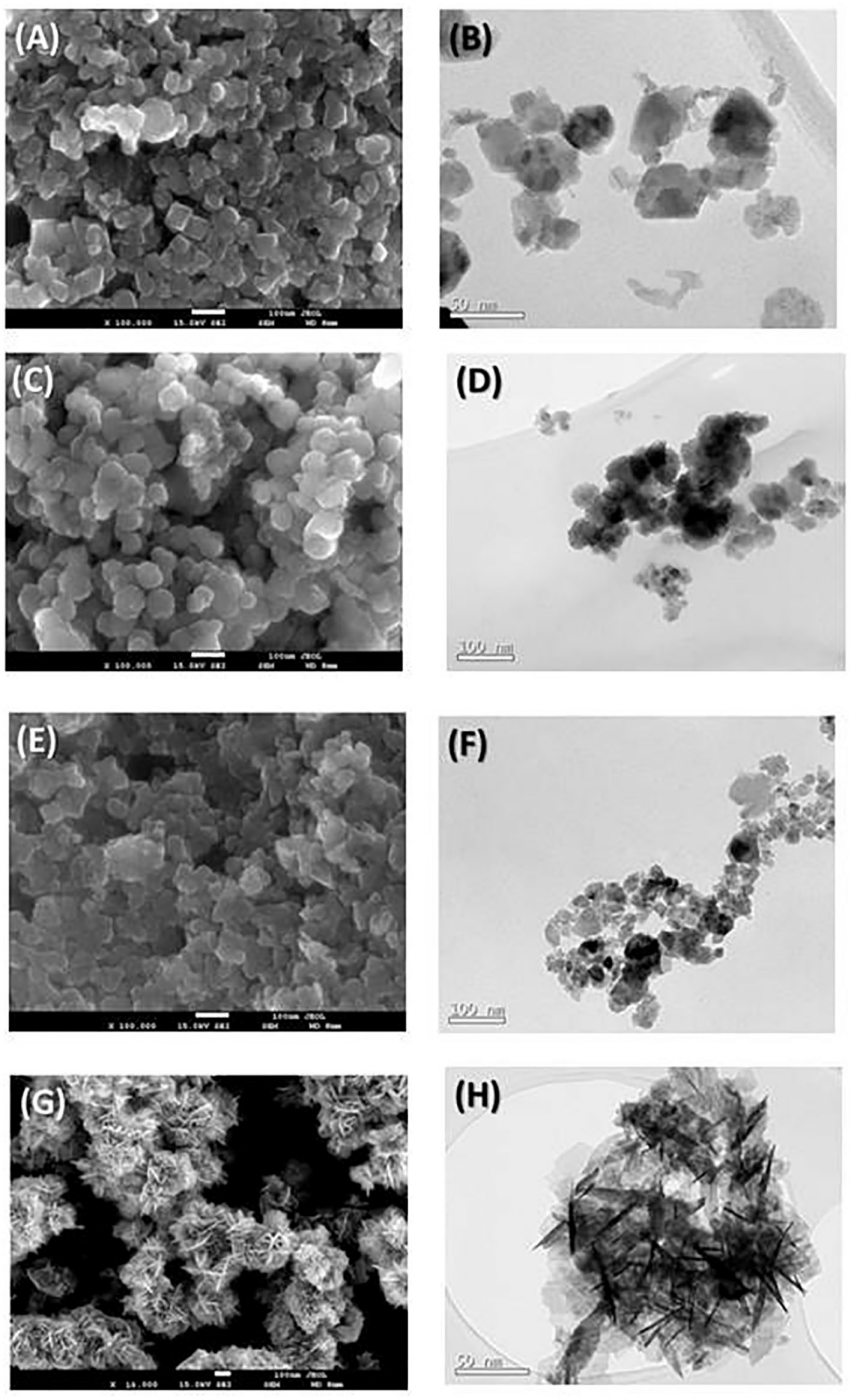
SEM (left) and TEM (right) micrographs for the prepared **(A,B)** L100, **(C,D)** L200, **(E,F)** L300, and **(G,H)** ZnO samples.

Figure 3 shows the SEM and TEM images of the obtained Zn/Mn oxide samples. In all the SEM micrographs, agglomerated particles with near-spherical morphologies are observed. Although some tetragonal crystals are visible in the SEM image of the L100 sample (with its higher Mn content), no changes in the morphology are observed with the variation of the stoichiometry. These polyhedral shapes are characteristic of the spinel phase, which has previously been reported for samples with similar compositions (Bordeneuve et al., 2010; Said and Harbrecht, 2019). A spherical particle shape is also observed in the TEM images. The average particle sizes were calculated from measurements of up to 80 particles. For the L100, L200, and L300 samples, the average sizes are 50, 40, and 35 nm, respectively. These results are in good agreement with previous studies (Thakur et al., 2008; Hadžić et al., 2016), in which an increase in the particle size was found with the Mn content in the samples. In the case of the ZnO sample (Figure 3G), rod-like structures that give rise to a flower-shaped morphology can be clearly observed. This particle shape is also observed in the TEM micrograph (Figure 3H) and is characteristic of ZnO samples, which was previously reported by several authors for samples obtained by sonochemical (Mishra et al., 2010), sol-gel (Kadhim et al., 2016), or solution (Wang et al., 2016) methods.

Both the XRD and microscopic characterizations indicate that, in the case of the Zn_*x*_Mn_3−x_O_4_ oxides, real mixed oxides were formed with only one type of morphology. This was not the case for the studies carried out by Pung et al. (2016), in which a ZnO–MnO_2_ core–shell nanocomposite was formed, or by Wang et al. (2019), in which both oxides were mixed to form a composite material that maintained both structures. In the study reported by Li et al. (2008a), the effects of the presence of MnO_2_ on the photocatalytic degradation of phenol by TiO_2_ and ZnO were investigated. In those cases, both MnO_2_ and ZnO were present, which is not the case in this study, wherein the mixed synthesis of a spinel structure was achieved.

### Optical Properties

**Figure 4:**
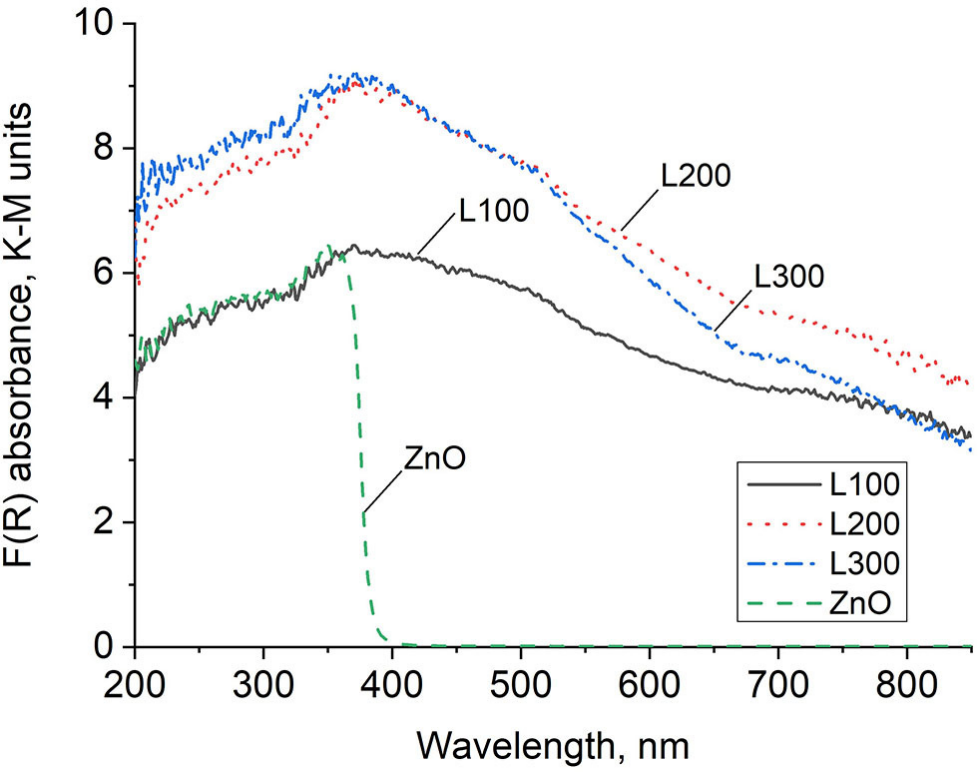
DRS absorbance spectra in Kubelka–Munk units.

Figure 4 shows the DRS spectra of the as-prepared samples. ZnO exhibits a continuous absorption band in the UV region, with a clear band edge at 365 nm, and does not absorb light in the visible region. Thus, the ZnO-recycled sample in this study shows typical ZnO semiconductor behavior (Zeng et al., 2014; Guzmán-Carrillo et al., 2017). The different Zn_*x*_Mn_3−x_O_4_ samples absorb in the UV region as well as steadily from 360 to beyond 800 nm. Thus, these samples show substantial absorption in the visible region, although clear band edges are not observed.

**Figure 5:**
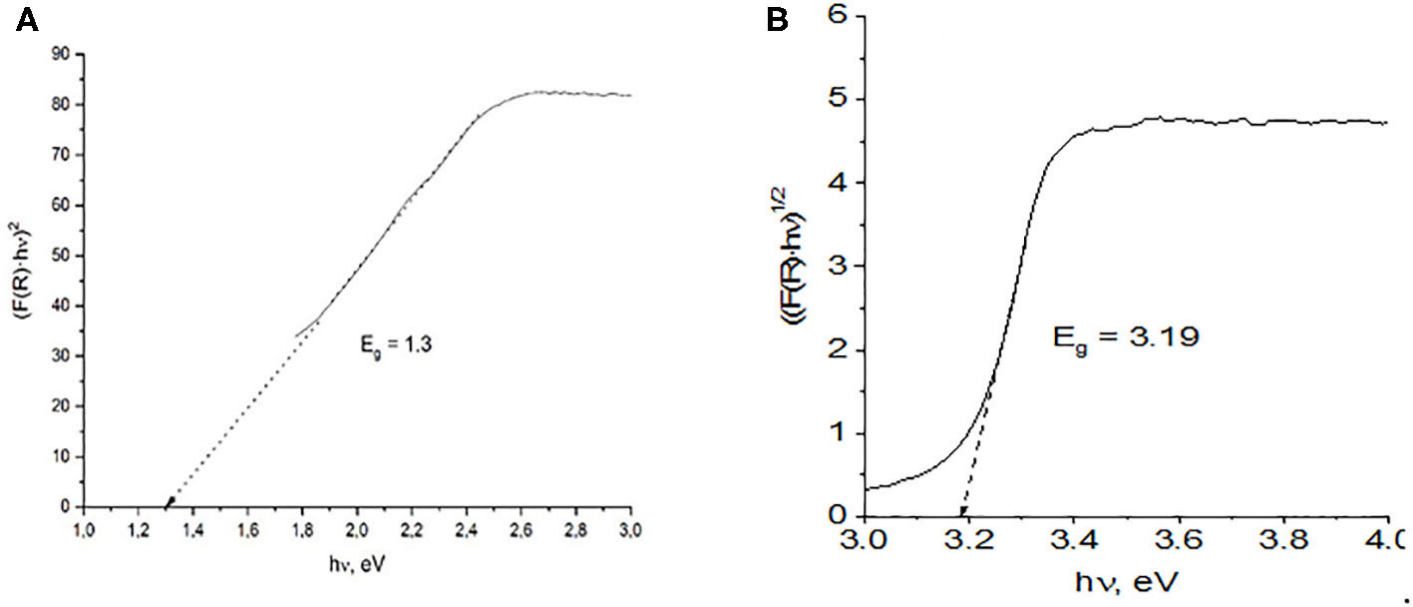
Band gaps estimated from the plot of the transformed Kubelka–Munk function vs. photoenergy for direct and indirect transitions of **(A)** L100 and **(B)** ZnO samples.

To obtain the band edge (*E*_g_) values for the obtained samples, the Kubelka–Munk optical absorption coefficients [*F*(R)] were plotted using Tauc's relation (Equation 4):

(4) $$\begin{array}{r} F\left(\text{R}\right)h\upsilon ={\left(h\upsilon -E_{\text{g}}\right)}^{n} \end{array}$$

where *h*ν represents the energy of the incident photon, and exponent *n* depends on the type of transition, with assigned values of 2 and 1/2 for indirect allowed and direct allowed transitions, respectively. Thus, the plot (*F*(R)*h*ν)^1/n^ vs. *h*ν follows a linear dependence in the region of the VB to the CB transition, and linear extrapolation to the abscissa gives the *E*_g_ value (Zeng et al., 2014; Guzmán-Carrillo et al., 2017). Figure 5 shows examples of the Tauc plots obtained from the DRS for the L-100 and ZnO samples for direct and indirect transitions, respectively. In the case of the mixed oxide, no intercept with the abscissa is found for *n* = 2, suggesting that the prepared Zn/Mn oxide only allows direct transitions at these energy levels. The values obtained for every sample for both types of transitions are listed in Table 3, which shows that the calculated band gap values for the Zn/Mn samples increase as the Mn content decreases. These results are in agreement with those previously reported for samples with similar stoichiometries (Zhang et al., 2011; Morán-Lázaro et al., 2018). The values correspond to band edges around 900 nm in the infrared region. The values found for ZnO in the UV region are also consistent with data reported elsewhere (Srikant and Clarke, 1998; Zhang et al., 2009; Ramya et al., 2019).

**Table 3:** Band gap energies estimated from plots of the transformed Kubelka–Munk function vs. photoenergy for indirect and direct transitions of all samples.

**Sample**	**Band gap**, ***E***_****g****_ **(eV)**	
	**Direct**	**Indirect**
L100	–	1.30
L200	–	1.35
L300	–	1.38
ZnO	3.19	3.27

The formation of unique structures including both Zn and Mn compounds, previously demonstrated by the microstructural characterization images (Figure 3), is also reflected in the DRS spectra results (Figure 4). These reveal a clear band edge for ZnO in the UV region, originating from the electron transition from the VB to the CB. The Zn/Mn oxides present DRS spectra similar to those of pure MnO_2_ samples, with strong absorption in the UV region and a continuous absorption band in the Vis region, where a clear band edge cannot be observed. This indicates that these mixed oxides do not show typical semiconductor behavior (Li et al., 2008b). Additionally, these samples are deep brown in color, which increases the spectral absorbance intensity (Levinson et al., 2005; Laplaza et al., 2017).

**Figure 6:**
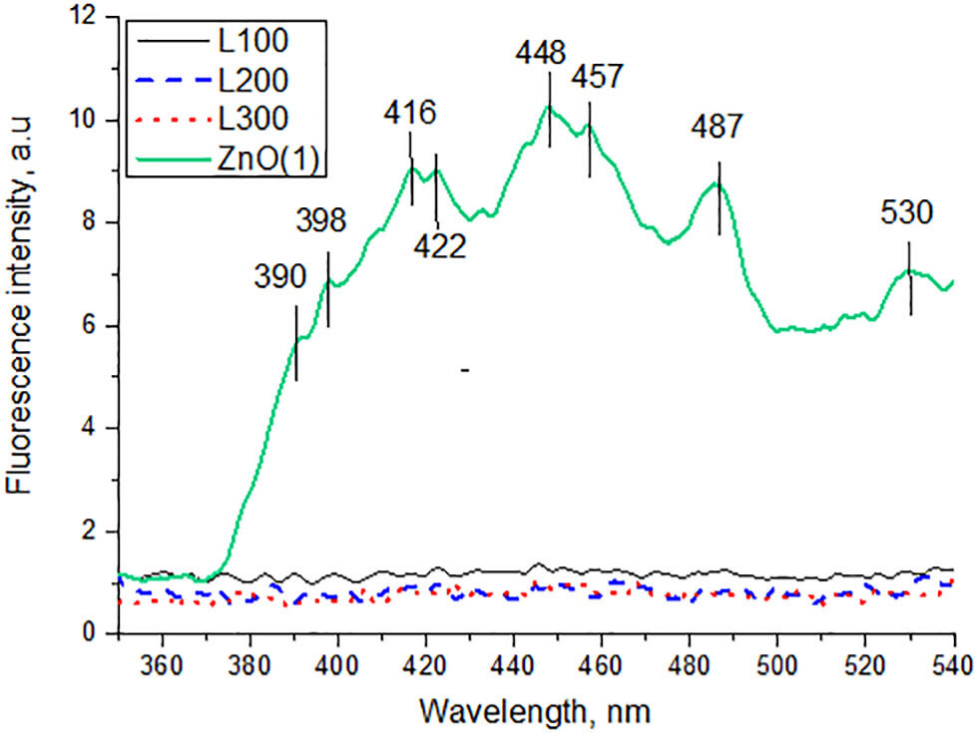
Photoluminescence spectra of samples excited at 325 nm.

Photoluminescence (PL) signals can provide valuable information about e^−^/h^+^ recombination processes in the main energy bands or defect states of a material. Figure 6 shows the PL spectra of the samples at an excitation wavelength of 325 nm. Only ZnO, which shows the typical spectrum of a semiconductor (see Figure 4), exhibits broad PL signals in the wavelength range 350–550 nm. In contrast, the Zn_*x*_Mn_3−x_O_4_ samples emit no signals.

The PL spectrum of the ZnO sample exhibits a broad emission band in the UV region (<400 nm), which is attributed to a near band edge transition (Wu et al., 2009). The other emissions in the visible region are presumably due to e^−^/h^+^ recombination at a deep-level emission in the band gap caused by intrinsic defects. Vanheusden et al. (1995) reported that the luminescence of ZnO mainly originates from defect states such as zinc interstitials and oxygen vacancies. The violet emission peaks observed between 416–422 and 448–457 nm are attributed to the presence of zinc interstitials. A transition involving a zinc vacancy produces a blue emission, which may correspond to our 487 nm peak. The green emission band at 530 nm is usually considered a deep-level or trap-state emission.

### Photocatalytic Activity Tests

#### Resazurin (Rz)-Resorufin Dye Test

**Figure 7:**
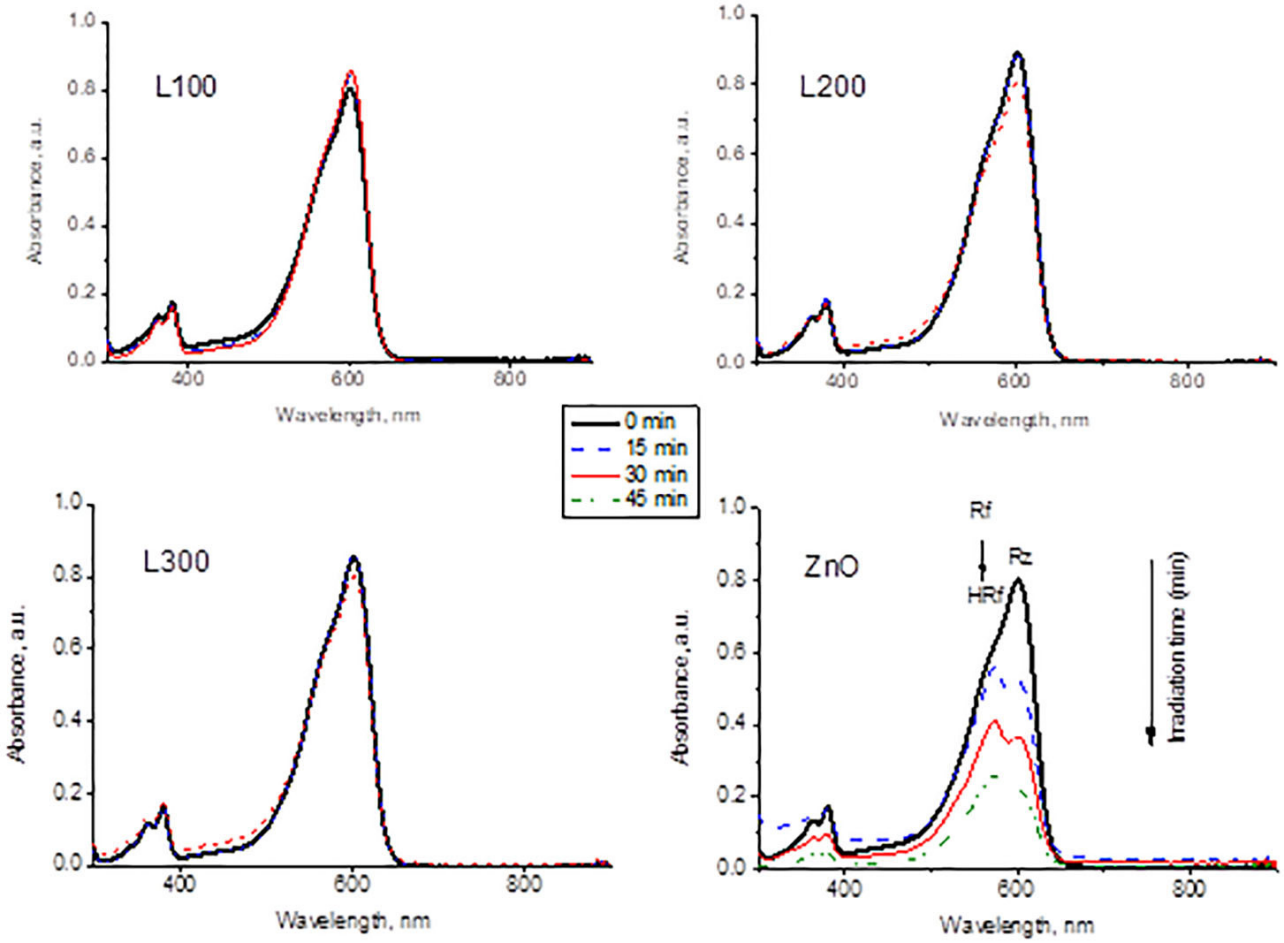
Variation of Rz dye spectra as a function of UV irradiation time in contact with the prepared samples.

Figure 7 presents the variations in UV–Vis absorbance of the Rz dye under UV irradiation for each sample. The absorption intensity of Rz declines significantly as a function of irradiation time only for the ZnO sample. This sample is able to partially transform Rz into Rf and subsequently bleaches the ink by Rf degradation to its colorless counterpart HRf. In contrast, the dyes in the presence of the Zn_*x*_Mn_3−x_O_4_ samples do not show any changes in absorbance during irradiation, indicating the lack of photocatalytic activity for these samples.

#### TA-FL Probe Method: Hydroxyl Radical (OH_·_) Generation

**Figure 8:**
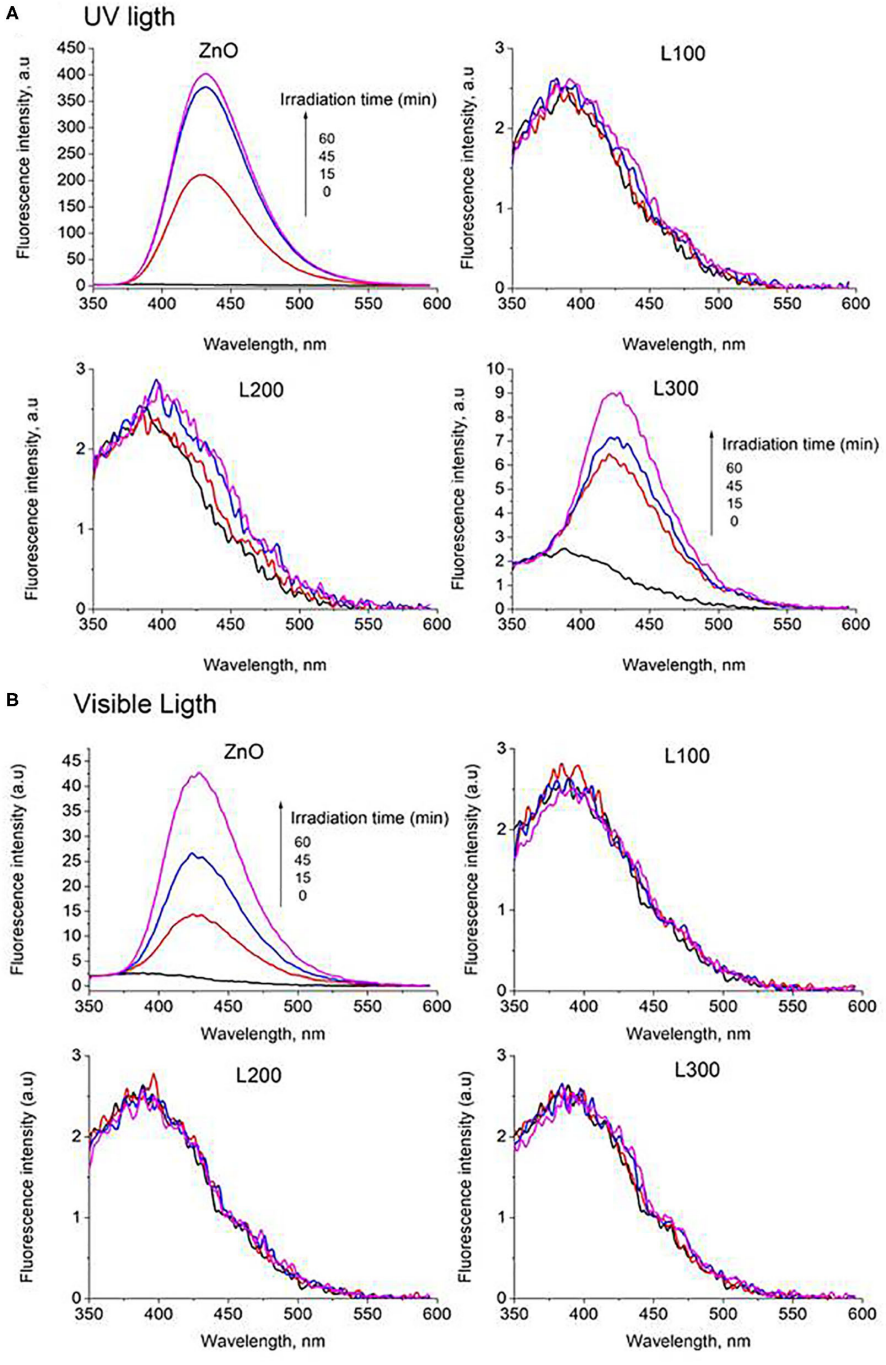
Fluorescence intensity spectra of all samples under **(A)** UV and **(B)** Vis light.

**Figure 9:**
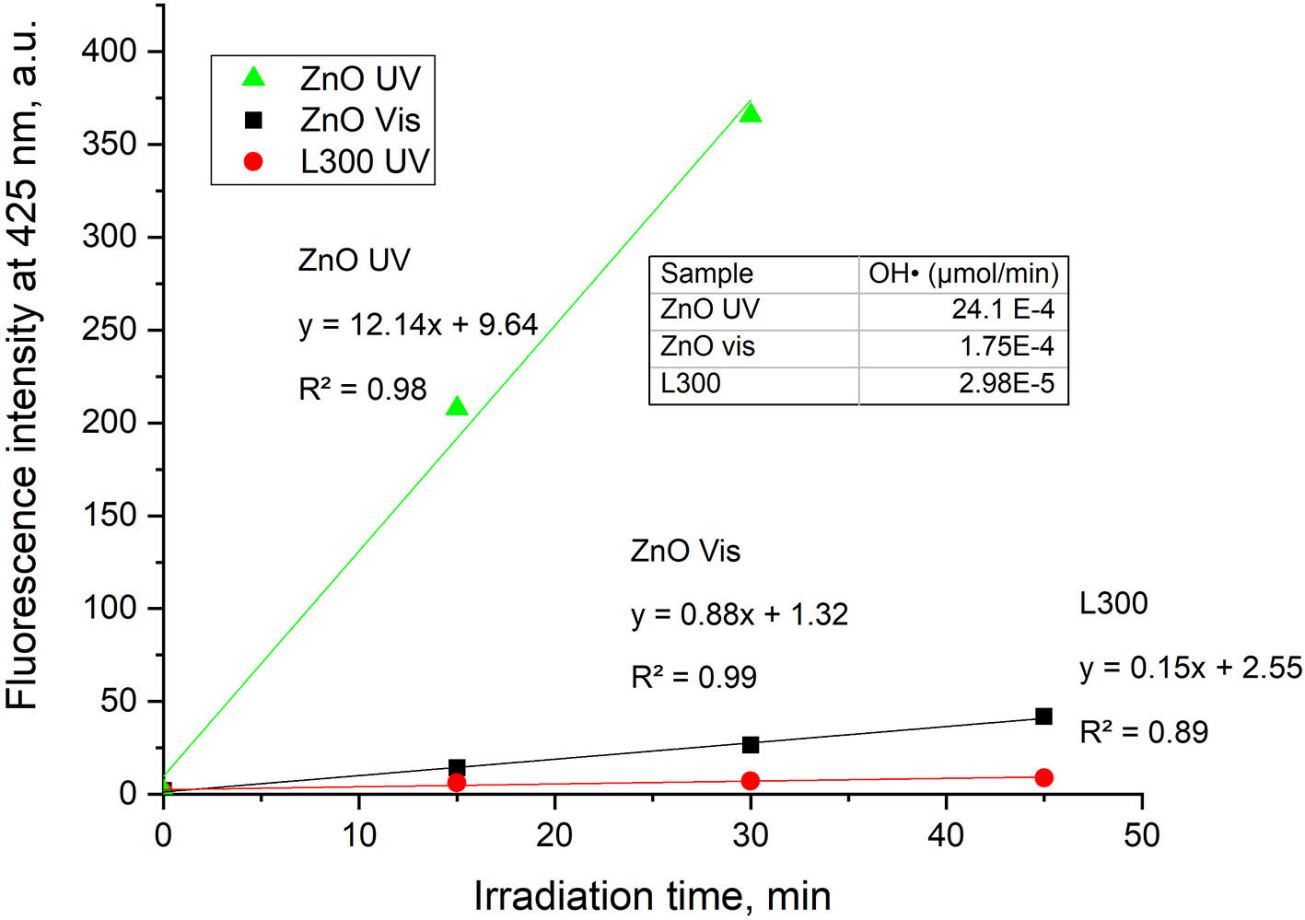
FL intensity at 425 nm vs. time of irradiation for the ZnO and L300 samples under UV and Vis irradiation (graph only includes the initial linear data). The inset table shows the rates of OH_·_ production/min.

The FL intensity spectra of the samples are illustrated in Figures 8A,B for UV and Vis light, respectively. The ZnO sample shows a significant increase in fluorescence at 425 nm under both UV and Vis irradiation. Therefore, it can be deduced that this sample generates OH_·_ in a photocatalytic process under both types of illumination. The OH_·_ formation is greater under UV light irradiation than under visible light. Provided that ZnO does not absorb visible light, the generation of OH_·_ might be ascribed to the presence of defects such as oxygen vacancies and zinc interstitials, in agreement with the PL results. Furthermore, sample L300 shows the slight formation of the fluorescent product TAOH under UV light. This may be related to the higher Zn concentration in its composition compared to the other Zn/Mn samples. Comparing the FL intensities at 425 nm as a function of irradiation time for ZnO and L300 in Figure 9, it is clear that the formation of TAOH for L300 is not significant in comparison with the pure ZnO sample, even though the L300 test was conducted with a sample amount that was one order of magnitude higher. The rates of OH_·_ formation (μmol/min) were calculated from the slopes in Figure 9.

#### Nitrogen Oxide Removal Test (ISO 22197-1:2007)

**Figure 10:**
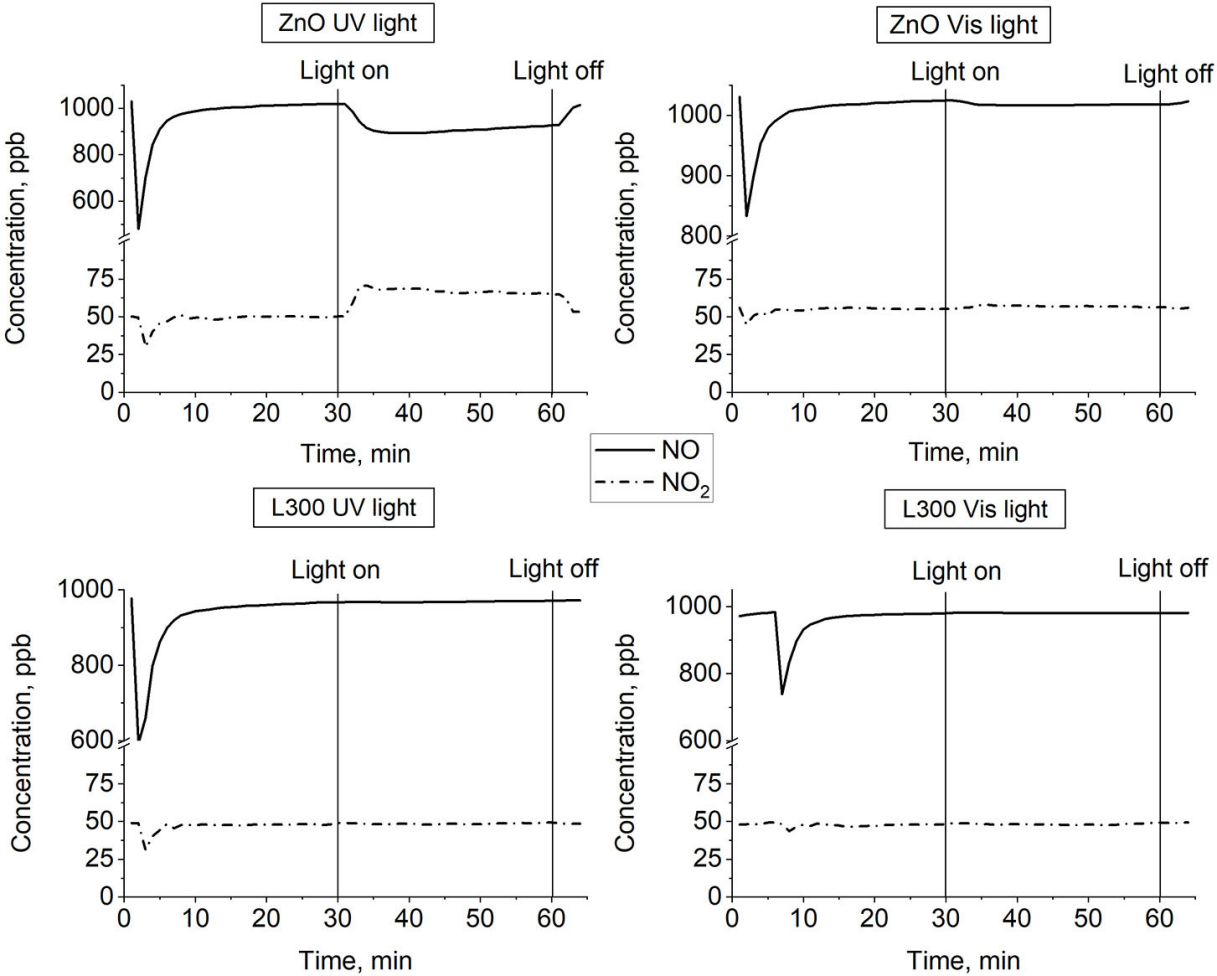
NO and NO_2_ concentration profiles vs. time for ZnO and L300 samples under UV and visible irradiation.

**Figure 11:**
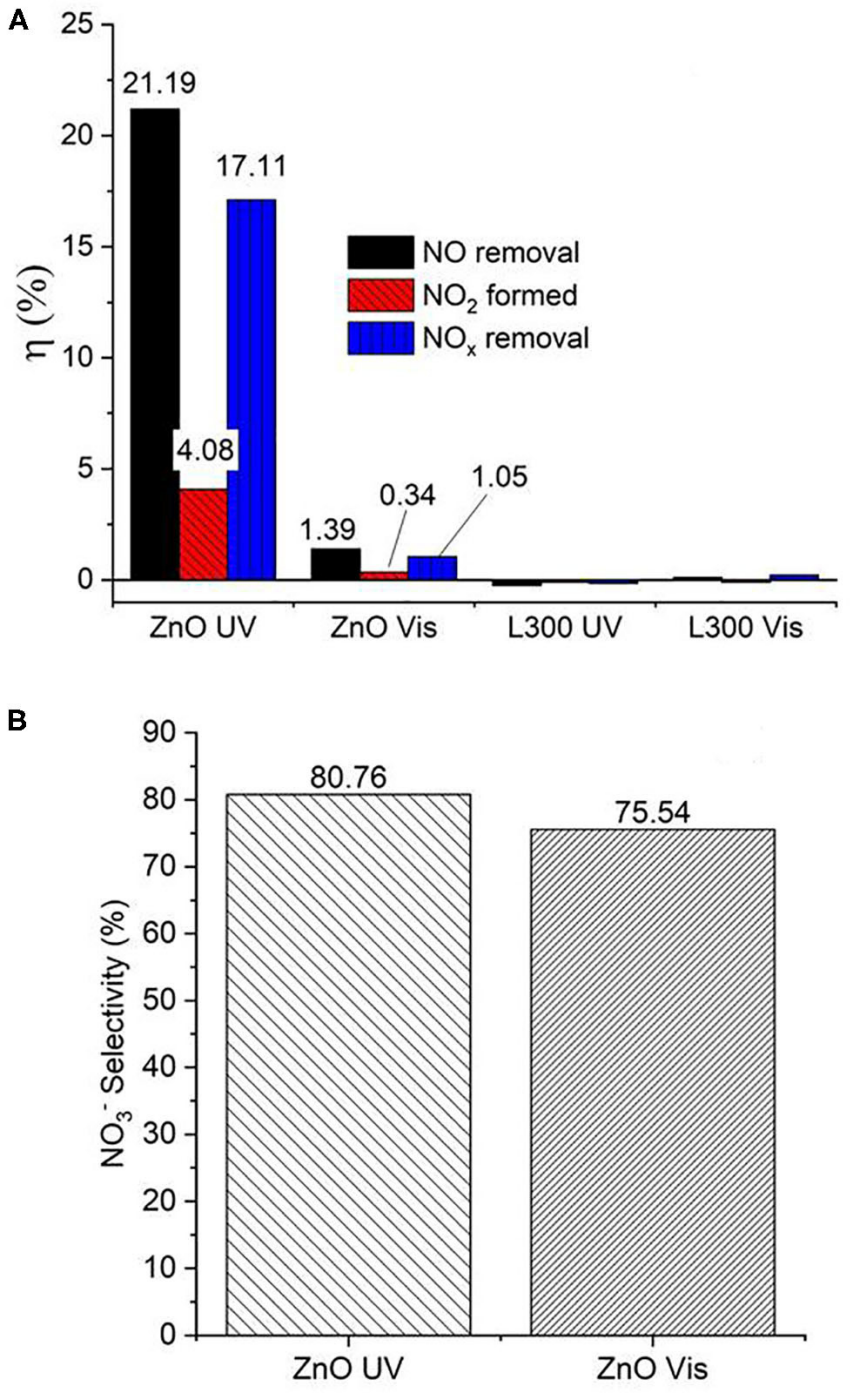
**(A)** Efficiencies (η, %) of NO/NO_x_ removal and NO_2_ formation by ZnO and the L300 composite. **(B)** Nitrate selectivity (%) of ZnO under both UV and Vis irradiation.

Figure 10 presents the NO and NO_2_ concentration profiles in the dark and under illumination in the presence of the prepared materials over time. Figure 11 shows a summary in terms of efficiency (η) of NO-NO_x_ removal, including the NO_2_ formed, for the ZnO and L300 samples. Only the ZnO sample exhibits photocatalytic activity under the test conditions. These results are consistent with other efficiency tests previously reported; a direct relationship between the TA-FL probe method and NO_x_ removal has previously been investigated (Jimenez-Relinque and Castellote, 2015; Laplaza et al., 2017). Additionally, the ${\text{NO}}_{3}^{-}$ selectivity (i.e., the ratio of NO converted to nitrates) was calculated according to Equation (5) (Bloh et al., 2014):

(5) $$\begin{array}{r} {\text{NO}}_{3\;}^{-}\text{selectivity }\left(\%\right)=\frac{\eta {\text{NO}}_{\text{Xremoval}}}{\text{ηNOremoved}}\cdot 100 \end{array}$$

This factor is very important, as NO_2_ is more toxic than NO, and better conversion to ${\text{NO}}_{3}^{-}$ is preferable for mitigating the negative global effects of NO_2_ on the environment and public health (Nava-Núñez et al., 2020). Remarkably, the ZnO sample shows acceptable nitrate selectivity under both irradiation types (although slightly lower by Vis).

In the present study, only the pure ZnO sample shows photocatalytic activity, while the Zn/Mn oxides demonstrate no photocatalytic performance. TA-FL probe test allowed obtaining a small indication of photocatalytic activity under UV light irradiation of the mix sample L300. This test is highly sensitive, enabling one to detect small quantities of OH_·_ generated on light-activated photocatalytic surfaces. However, it is not sufficient to give a detectable NO removal during the performed NO air purification test conditions. Furthermore, this photogeneration rate of active species does not seem enough to reduce the Rz to Rf. Note that the test using L300 was made with more than 10 times the amount of ZnO.

The poor performance results of Zn/Mn oxides can be attributed to different factors. Given the low energy difference between the VB and CB of MnO_2_, a rapid recombination process for the photogenerated e^−^/h^+^ pairs could occur. This recombination might be forced during the photocatalysis process, and the input energy dissipated in the form of light or heat. However, the PL measurements indicate that the Zn/Mn samples do not show any emission signal for e^−^/h^+^ recombination. Generally, the PL emissions of semiconductor materials originate from the radiative recombination of e^−^/h^+^, leading to UV-region emission signals. The second emission route is an initial transfer of e^−^ from the CB to different sub-bands such as surface oxygen vacancies or intrinsic defects via non-radiative transitions, which may subsequently transfer from the sub-bands to the VB via radiative transition with the release of PL signals. These corresponding PL signals can be observed in the visible region (Liqiang et al., 2006; Yan et al., 2013; Choudhury and Choudhury, 2014; Paul and Choudhury, 2014). In this study, the absence of e^−^/h^+^ recombination PL emission signals from the Zn/Mn samples in the range of light used can be explained by some e^−^ transfer from the CB to deep trapped sub-band states, or the relaxation of partial excitons that would be transferred by non-radiative paths and, therefore, do not emit PL signals (Wang et al., 2019). Generally, the charge carriers from the defects have short lifetimes that do not significantly contribute to photocatalysis (Cushing et al., 2017). On the other hand, it may also be that under UV and Vis, activation is more energetic than the IR band gap estimated for the Zn/Mn oxides and does not efficiently separate the charge carriers to produce photocatalytic processes, even though the materials show strong absorption in both region spectra. Although the confirmation of these hypotheses is beyond the scope of this paper, these ideas will be studied next by the authors.

The study of ZnO as photocatalyst in the oxidation reaction of NO_x_ is limited (Wei et al., 2013; Nava Núñez and Martínez-de la Cruz, 2018; Nguyen et al., 2019). Some of them are focused on enhancing titanium dioxide De-NO_x_ efficiency through the preparation of TiO_2_/ZnO composites (Pei and Leung, 2013; Wei et al., 2013; Giannakopoulou et al., 2014). The pure ZnO photocatalyst, whose preparation proceeded by using amine derivatives or ionic liquids, showed a NO conversion photo-efficiency equal to or lower than 50% (Kowsari and Bazri, 2014; Kowsari and Abdpour, 2017). On the other hand, ZnO synthetized routes in the presence of complex structuring agents have reached highest performances ~70–95%, even better than that of commercial ZnO (Morán-Lázaro et al., 2018; Nava-Núñez et al., 2020). However, the reported values of photocatalytic performance ZnO are strongly dependent on the morphology and the preferential crystalline orientation of its particles (Nava-Núñez et al., 2020). Furthermore, there are several variations in the procedure tests (irradiation characteristics, amount of sample, test time, NO_x_ gas concentration, etc.), which makes it difficult to make a comparative assessment across those results. In general, the NO conversion is higher using UV irradiation than Vis light, in good agreement with the obtained results in the present work (Wei et al., 2013). Interestingly, in this work, the ZnO nanoparticle photocatalysts are prepared from a waste product with difficult management, in our specific case, from black mass from spent alkaline batteries. The efficiency obtained under UV light (~21%) demonstrated that the conversion of this waste could lead to a useful air purification photocatalyst. The enhancement of this performance could convert these treated waste into a promising catalyst for industrial scale.

## Conclusions

Several oxides with Zn_*x*_Mn_3−x_O_4_ stoichiometries as well as ZnO were prepared from the black mass waste generated from spent alkaline batteries. The XRF results revealed Mn as the major element in all the mixed oxides. The XRD patterns for all the Zn/Mn oxides showed that the most intense diffraction peak could be indexed on the basis of a tetragonal symmetry in the *I*4_1_*amd* space group, which is consistent with a spinel-type structure. Moreover, Rietveld refinements and wt% values obtained from the XRF results suggested stoichiometries of Zn_*x*_Mn_3−x_O_4_ for the oxide samples. Morphological characterization showed agglomerated, nearly spherical particles, although some tetragonal crystals typical of spinel-type structures were observed in the SEM micrographs. Optical characterization indicated that ZnO had a clear band edge in the UV region, while the Zn/Mn oxides exhibited strong absorption in the UV region and a continuous absorption band in the Vis-IR regions. PL signals due to e^−^/h^+^ recombination were only detected for ZnO. The absence of PL emission signals for the Zn/Mn samples in the tested wavelength range was attributed to the trapping of excited charges in sub-band energy states and/or transfer by non-radiative paths. The lack of photocatalytic activity for the Zn/Mn oxides can also be variously attributed, such as to a higher recombination rate due to the low band gap of MnO_2_ or the presence of deep sub-band gap trapped defects. Generally, the charge carriers from defects have short lifetimes and may not significantly contribute to photocatalysis. On the other hand, it is possible that under the UV–Vis light, activation is more energetic than the IR band edge estimated for the Zn/Mn oxides and does not efficiently separate the charge carriers to promote the photocatalytic process.

## Data Availability Statement

The raw data supporting the conclusions of this article will be made available by the authors, without undue reservation.

## Author Contributions

FL and MC conceived the study. LA, EJ-R, IG-D, and LP carried out the experiments. LA, EJ-R, and LP wrote the manuscript. All authors contributed to the review, editing, and approval of the paper.

## Conflict of Interest

The authors declare that the research was conducted in the absence of any commercial or financial relationships that could be construed as a potential conflict of interest.
